# Correction: Socioeconomic position and the influence of food portion size on daily energy intake in adult females: two randomized controlled trials

**DOI:** 10.1186/s12966-023-01492-4

**Published:** 2023-08-04

**Authors:** Tess Langfeld, Katie Clarke, Lucile Marty, Andrew Jones, Eric Robinson

**Affiliations:** 1 Department of Psychological Sciences, University of Liverpool, Liverpool, UK; 2 Centre Des Sciences Du Goût Et de L’Alimentation, CNRS, INRAEInstitut AgroUniversité Bourgogne Franche-Comté, 21000 Dijon, France

**Correction: Int J Behav Nutr Phys Act 20: 53 (2023)**

**https://doi.org/10.1186/s12966-023-01453-x**

Following publication of the original article [1], the authors identified an error in Fig. 2. The correct figure is given below.

**Fig. 2:**
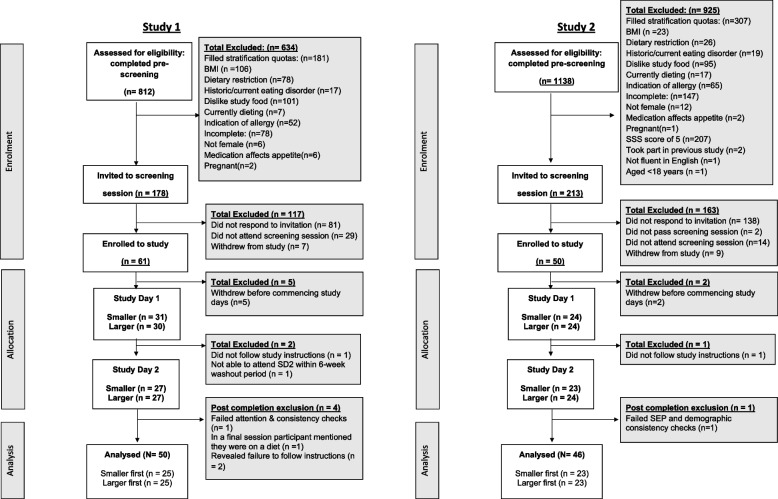
CONSORT flowchart for participant enrolment, allocation, and analysis for Study 1 (left panel) and Study 2 (right panel). Figure legend. Attention checks were included in online questionnaires (e.g., “When did you last visit the Moon”). Consistency checks were also included in online questionnaires (e.g., verifying highest educational qualification)

The original article [1] has been corrected.
