# Supplementary material for: Socioeconomic position and the influence of food portion size on daily energy intake in adult females: two randomized controlled trials
Source: Int J Behav Nutr Phys Act. 2023 Apr 27;20:53. doi: 10.1186/s12966-023-01453-x (PMC10134633; doi:10.1186/s12966-023-01453-x)
Supplement: Supplementary file 1 — Additional file 1. Table S1. Dishware used in the present study. Table S2a. Full Study Menus – STUDY 1. Table S2b. Full Study Menus – STUDY 2. Table S3. Nutritional value of study foods (per 100g). Table S4. Educational equivalents. Table S5. Secondary analysis - mixed ANOVA results predicting total daily energy intake (kcal): portion size, individual difference measure, and portion size * individual difference measure. Table S6. Sensitivity analysis - mixed ANOVA results predicting total daily energy intake (kcal): portion size, alternative SEP indicator, and portion size * SEP indicator. Table S7. Mean (SD) Normality, Familiarity and Liking ratings of all portion-manipulated study foods. Table S8. Counts of participants who reported noticing a difference in portion size (yes/no) and who guessed the portion sizes of each meal correctly (yes/no). Table S9. Counts of participants who reported noticing a difference in portion size and accurately guessed the portion sizes split by SEP group. [file 12966_2023_1453_MOESM1_ESM.docx]

**Supplementary Information for:**

Socioeconomic position and the influence of food portion size on daily energy intake in adult females: two randomized controlled trials

**Author names and affiliations:**

Tess Langfield ^1^, Katie Clarke ^1^, Lucile Marty ^2^, Andrew Jones ^1^ & Eric Robinson ^1^

^1^ Department of Psychological Sciences, University of Liverpool, Liverpool, UK

^2^ Centre des Sciences Du Goût et de l’Alimentation, CNRS, INRAE, Institut Agro, Université Bourgogne Franche-Comté, F-21000 Dijon, France

**Corresponding author information:**

Eric Robinson, Department of Psychological Sciences, University of Liverpool, Eleanor Rathbone Building, Bedford Street South, Liverpool L69 7ZA, UK, +44 151 794 1187, [eric.robinson@liverpool.ac.uk](mailto:eric.robinson@liverpool.ac.uk)

**Additional methodological information**

**Participants and recruitment**

In Study 1, we pre-registered recruitment of participants with a BMI of between 22.5-32.5kg/m^2^ because approximately 70% of adults in England fall within this band (1). Due to difficulties in recruitment, this was adjusted to 18.5-32.5kg/m^2^. In Study 2, we pre-registered recruitment of participants with a BMI of between 18.5 – 32.5kg/m^2^. For the same reasons this was adjusted to 18.5 – 39.9kg/m^2^ during data collection. Note: SEP groups did not differ on BMI in either study and we found no evidence in analyses that BMI moderated the effect of portion size on energy intake in either study. Because students disproportionately represented the higher SEP group in Study 1, in Study 2 recruitment was also stratified by student status (4.1% yes, broadly representative of the UK adult population; (2)). In Study 2 we also stratified recruitment by SSS score to ensure that the majority of participants recruited did not cluster close to the mid-point (5) of the SSS scale: no more than 50% of could score ‘6’ (higher SSS) or ‘4’ (lower SSS) to maximise SSS differences by group (this resulted in participants scoring 1-3 and 7-10 on the SSS scale being represented in the study). Eligibility was checked using an online questionnaire and then confirmed during an in-person screening session. Eligibility checks included checking participant responses against exclusion/inclusion criteria and study quotas to meet stratified recruitment requirements.

**Menu choices**

**Study 1.** We selected foods that have been used in similar studies and palatable for the study population (e.g., (3)). We aimed to choose portion sizes that would be perceived as ‘normal’ in order to limit potential substantial compensatory eating after consuming a meal perceived as being ‘smaller than normal’. In particular, we selected smaller and larger portions based on previous research which found the ‘norm-range’ for several dishes (i.e. the range of portions perceived as ‘normal’ by at least 60% of a given sample; (3)).

**Study 2.** The menus were adapted from Study 1. To reduce food waste, based on data on intake from Study 1, the amounts of food available at lunch were reduced (see Table 1 of main manuscript). Based on feedback from participants in Study 1, the breakfast menu was diversified (by adding a brown bread option, and yoghurt). To boost recruitment by allowing vegetarians to take part, the beef chilli was swapped for a vegetarian chilli (portion sizes (g) kept the same), with an additional side added (tortilla chips) and the vegetable side swapped from sweetcorn to mixed vegetables to increase variety. Finally, again to reduce food waste and based on data on dessert intake from Study 1, the number of units of brownie bites and millionaire shortbread bites offered in the ad-libitum dessert buffet was reduced from 10 to 5 (for each).

**Dishware**

In Study 1, dishware size was varied across study session days at breakfast (smaller vs larger). Participants were provided a breakfast buffet including toast, cereal, jam, butter, and milk. They were provided a plate and a bowl onto which to serve their food, which varied in size (smaller vs larger; see Table S1 for further details about the dimensions of dishware used).

**Table S1. Dishware used in the present study**

|  |  | **Diameter (cm)** | **Surface area (cm^2^)** | **Volume capacity (ml)** |
| --- | --- | --- | --- | --- |
| **Bowl** | **Smaller** | 15 | - | 360 |
|  | **Larger** | 18 | - | 550 |
| **Plate** | **Smaller** | 16.5 | 213.8 | - |
|  | **Larger** | 22.8 | 408.3 | - |

Manipulation of dishware size (smaller followed by larger dishware size vs larger followed by smaller dishware size) was counterbalanced with the order of portion size presentation (smaller followed by larger portions vs larger followed by smaller portions). There was no evidence that dishware size impacted energy (kcal) intake at breakfast (p>.05) and controlling for dishware size condition did not affect the results of the primary analyses. Results relating to dishware size will be detailed in a separate report. For further information see: <https://osf.io/apxnh/>.

|  | **Portion**  **(g)** | **Energy content (kcal)** | **Liking**  **(mean, SD)** |
| --- | --- | --- | --- |
| **Breakfast** |  |  |  |
| **White toast (4 pieces)** | 160 | 390 | 5.14 (1.71) |
| **Cornflakes** | 120 | 464 | 3.98 (1.77) |
| **Milk** | 500 | 250 | - |
| **Strawberry jam** | 60 | 148 | - |
| **Sunflower spread** | 40 | 168 | - |
| **Snack box** |  |  |  |
| **Apples (2)** | 268 | 142 | 5.00 (2.10) |
| **Cereal bars (2)** | 60 | 233 | 4.52 (2.02) |
| **Crisps (2 packs)** | 50 | 270 | 4.56 (2.07) |
| **Carrot sticks** | 100 | 43 | 3.96 (2.26) |
| **Biscuits (2)** | 22 | 105 | 4.62 (2.26) |
| **Fruit and nut mix** | 25 | 117 | 4.32 (2.22) |
| **Lunch** |  |  |  |
| **Cheese and tomato pasta bake** |  |  | 5.42 (1.39) |
| **SMALLER** | 375 | 544 | - |
| **LARGER** | 563 | 816 | - |
| **Amount of additional servings available** | 750 | 1088 | - |
| **Dinner** |  |  |  |
| **Beef chilli with rice** |  |  | 5.68 (1.32) |
| **SMALLER** | 291 | 339 | - |
| **LARGER** | 437 | 509 | - |
| **Amount of additional servings available** | 233 | 271 | - |
| **Sweetcorn** | 80 | 62 | - |
| **Cheese** | 40 | 166 | - |
| **Dessert** |  |  |  |
| **Chocolate brownie bites (10)** | 110 | 433 | 4.94 (2.09) |
| **Millionaire shortbread bites (10)** | 125 | 545 | 5.10 (2.01) |
| **Chocolate chip muffins (5)** | 110 | 550 | 4.54 (2.08) |

**Table S1. Full Study Menus – STUDY 1**

*Note.* Total energy available in smaller condition: 6328kcal. Total energy available in larger condition: 6770kcal. 500ml water available with each meal, and choice of tea or coffee and sugar on request, available with breakfast. Mean liking rated from 1 (Not at all) – 7 (Very much). Dashes indicate meal components which were not rated.

**Table S2. Full Study Menus – STUDY 2**

| **Food item** | **Portion**  **(g)** | **Energy content (kcal)** | **Liking (mean, SD)** |
| --- | --- | --- | --- |
| **Breakfast** |  |  |  |
| **White toast^a^** |  |  | 5.68 (1.51) |
| **SMALLER (2 PIECES)** | 80 | 195 | - |
| **LARGER (3 PIECES)** | 120 | 293 | - |
| **Brown toast** |  |  | - |
| **SMALLER (2 PIECES)** | 80 | 185 | - |
| **LARGER (3 PIECES)** | 120 | 277 | - |
| **Cornflakes** |  |  | 4.63 (1.69) |
| **SMALLER** | 30 | 116 | - |
| **LARGER** | 45 | 174 | - |
| **Yoghurt** |  |  | 5.26 (1.97) |
| **SMALLER** | 100 | 95 | - |
| **LARGER** | 150 | 143 | - |
| **Milk** | 500 | 250 | - |
| **Strawberry jam (5 sachets)** | 80 | 191 | - |
| **Butter (5 sachets)** | 35 | 260 | - |
| **Sugar** | 40 | 160 | - |
| **Snack box** |  |  |  |
| **Apples (2)** | 268 | 142 | 6.11 (1.26) |
| **Cereal bars (2)** | 60 | 233 | 5.43 (1.72) |
| **Crisps (2 packs)** | 50 | 270 | 5.41 (1.56) |
| **Carrot sticks** | 100 | 43 | 4.81 (1.78) |
| **Biscuits (2)** | 22 | 105 | 5.46 (1.77) |
| **Fruit and nut mix** | 25 | 133 | 5.35 (1.99) |
| **Lunch** |  |  |  |
| **Cheese and tomato pasta bake** |  |  | 5.13 (1.62) |
| **SMALLER** | 350 | 508 | - |
| **LARGER** | 525 | 761 | - |
| **Amount of additional servings available** | 280 | 406 | - |
| **Dinner** |  |  |  |
| **Vegetarian chilli with rice** |  |  | 5.43 (1.52) |
| **SMALLER** | 291 | 296 | - |
| **LARGER** | 437 | 444 | - |
| **Amount of additional servings available** | 233 | 237 | - |
| **Mixed vegetables** | 80 | 53 | - |
| **Cheese** | 40 | 166 | - |
| **Tortilla chips** | 30 | 150 | - |
| **Dessert** |  |  |  |
| **Chocolate brownie bites (5)** | 55 | 217 | 5.70 (1.60) |
| **Millionaire shortbread bites (5)** | 125 | 545 | 5.62 (1.77) |
| **Chocolate chip muffins (5)** | 55 | 275 | 4.91 (1.82) |

*Notes.* Total energy available in smaller condition: 5040kcal. Total energy available in larger condition: 5643kcal. 500ml water available with each meal, and choice of tea or coffee available with breakfast. Mean liking rated from 1 (Not at all) – 7 (Very much). Dashes indicate meal components which were not rated. ^a^ Note rating was for “Toast” rather than “White toast” and “Brown toast”

|  | **Energy** | **Fat**  **(total)** | **Fat (saturates)** | **Carbohydrate (total)** | **Sugars** | **Fibre** | **Protein** | **Salt** |
| --- | --- | --- | --- | --- | --- | --- | --- | --- |
| **White sliced bread** | 1025kJ | 2.0g | 0.5g | 46.4g | 3.0g | 3.0g | 9.1g | 0.98g |
| **Brown sliced bread^b^** | 974kJ | 2.8g | 0.5g | 37.8g | 2.4g | 6.4g | 10.6g | 0.95g |
| **Cornflakes** | 1641kJ | 1.2g | 0.3g | 85.2g | 6.1g | 2.5g | 7.7g | 0.59g |
| **Semi-skimmed milk** | 209kJ | 1.8g | 1.1g | 4.8g | 4.8g | 0.0g | 3.6g | 0.1g |
| **Raspberry yoghurt^b^** | 399kJ | 2.7g | 1.7g | 13g | 12g | 0.5g | 3.8g | 0.24g |
| **Strawberry jam** | 1048kJ | 0.4g | 0.2g | 59.8g | 44.6g | 1.7g | 0.2g | 0.1g |
| **Sunflower spread^a^** | 1732kJ | 45.7g | 11.8 | 2.1g | 0.1g | 0.5g | 0.1g | 1.45g |
| **Dairy butter^b^** | 3059kJ | 82g | 52g | 0.6g | 0.6g | 0g | 0.6g | 1.7g |
| **Sugar** | 1700kJ | 0g | 0g | 100g | 100g | 0g | 0g | 0g |
| **Chocolate and vanilla biscuits** | 1997kJ | 20g | 5.4g | 68g | 38g | 2.7g | 5.3g | 0.73g |
| **Apples** | 226kJ | 0.1g | <0.1g | 11.8g | 11.8g | 1.8g | 0.4g | <0.01g |
| **Carrot sticks** | 175kJ | 0.3g | 0.1g | 7.4g | 7.0g | 3.9g | 0.4g | 0.07g |
| **Cereal bars** | 1636kJ | 7.1g | 0.9g | 72.9g | 29.2g | 4.0g | 6.0g | 0.03g |
| **Fruit and nut mix** | 1955kJ | 24.9g | 3.2g | 44.5g | 40.3g | 5.8g | 13.6g | 0.01g |
| **Ready salted crisps** | 2250kJ | 33.6g | 3.2g | 51.1g | 0.5g | 4.8g | 5.8g | 1.1g |
| **3-cheese pasta bake** | 548kJ | 3.1g | 1.7g | 18.8g | 2.8g | 1.7g | 5.9g | 0.25g |
| **Beef chilli^a^** | 370kJ | 2.7g | 1.0g | 8.2g | 2.5g | 2.6g | 6.4g | 0.42g |
| **Vegetarian chilli^b^** | 321kJ | 2.2g | 0.2g | 9.1g | 3.1g | 3.6g | 3.3g | 0.35g |
| **Long-grain rice (cooked)** | 578kJ | 1.4g | 0.3g | 28.0g | 0.1g | 1.0g | 2.5g | 0.1g |
| **Sweetcorn^a^** | 321.25 | 1.4g | 0.3g | 11.8g | 5.1g | 2.6g | 2.8g | 0.1g |
| **Mixed vegetables^b^** | 245kJ | 0.8g | 0.1g | 7.9g | 4.1g | 4.4g | 2.6g | 0.03g |
| **Cheddar cheese** | 1725kJ | 34.9g | 21.7g | 0.1g | 0.1g | 0g | 25.4g | 1.8g |
| **Lightly salted tortilla chips^b^** | 2090kJ | 23.5g | 2.0g | 64.9g | 1.0g | 2.9g | 5.5g | 0.80g |
| **Chocolate chip muffins** | 1823kJ | 22.5g | 3.6g | 52.5g | 28.4g | 1.6g | 5.0g | 0.34g |
| **Millionaire shortbread bites** | 2089kJ | 28.0g | 15.2g | 55.6g | 36.6g | 2.5g | 5.1g | 0.50g |
| **Chocolate brownie bites** | 1655kJ | 15.0g | 6.6g | 58.0g | 36.8g | 3.1g | 5.3g | 0.4g |

Table S3. Nutritional value of study foods (per 100g)

Note. ^a^ Study 1 only. ^b^ Study 2 only.

**Socioeconomic and demographic measures**

**Highest educational qualification.** Highest educational qualification was assessed by asking participants: *“What is the highest educational qualification that you have received or are currently studying for?”*. Responses were coded from 1 to 9 (1 = No formal qualifications; 2 = 1–3 GCSEs; 3 = 4+ GCSEs; 4 = A level; 5 = Certificate of higher education (CertHE); 6 = Diploma of higher education (DipHE); 7 = Bachelor; 8 = Master’s degree; 9 = Doctorate). Responses were categorised as a binary variable: lower (values: 1, 2, 3, 4; A level/equivalent or less) or higher (values: 5, 6, 7, 8, 9; qualifications above A level).

*
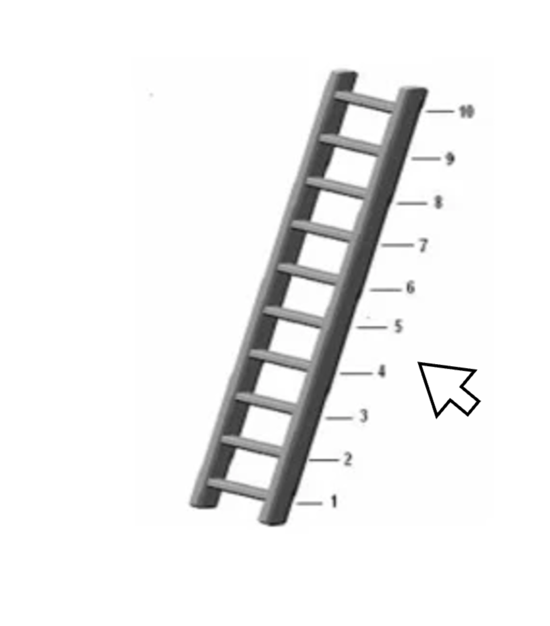
***Subjective social status (SSS).** SSS was assessed the MacArthur Scale. Figure below adapted from (4). Participants were asked: *“Think of a ladder (see image) as representing where people stand in society. At the top of the ladder, are the people who are best off — those who have the most money, most education and the best jobs. At the bottom, are the people who are worst off—who have the least money, least education and the worst jobs or no job. The higher up you are on this ladder, the closer you are to people at the very top and the lower you are, the closer you are to the bottom.*

*Please select where best represents where you think you stand on the ladder.”*

Scores were indicated by moving the cursor and selecting a number, with a box appearing when hovering over the number. Scores were rated from 1 (lower SSS) – 10 (higher SSS), and in Study 2 were categorised as lower (1 – 4) and higher (6 – 10), with participants scoring 5 not eligible.

**Level of education.** A composite score of level of education was created in order to examine if results were consistent when both highest educational qualification and time in higher education were considered (highest educational qualification and number of years in higher education z-scored and averaged). Number of years in higher education was measured by asking participants “*After leaving school (i.e. at 16 years old), how many further years of higher education (i.e. a formal course) did you study for? If you left school and did not go on to study further in higher education, your answer would be 0. If you left school and then studied for two years for A levels, your answer would be 2. If you completed A levels over two years and then also studied for a three-year undergraduate degree, your answer would be 5*”.

**Equivalised disposable income**. Participants were asked about household income: “*What is your annual after tax household income, including all earners in your household, in GBP (to the nearest £1000)? (range £ 0 - 999,999)*” and about household composition: “*Thinking about all of the people who live at your house, including you: How many adult(s) or children aged 14 and over live at your house?* *How many child(ren) under the age of 14 live at your house?*”. The OECD-modified equivalence scale was used to adjust household income taking into account household size and composition (5). Equivalised household income was calculated by dividing the after-tax household (including all earners to the nearest £1000) by the sum of the equivalence value of all the household members (1 = first adult; 0.5 = additional adult or child >14 years old; 0.3 = child aged 0-13 years old).

**Self-reported financial hardship in childhood.** The 3-item measure of resource availability during childhood developed in 2011 by Griskevicius and colleagues (6) was used to assess self-reported financial hardship in childhood. This measure has been used in previous studies investigating its relationship with obesity or eating behaviour (7, 8). Participants rated questions (e.g. “my family had enough money for things growing up”) from 1 (Strongly disagree) to 7 (Strongly agree). Responses were averaged, with lower scores indicating greater financial hardship in childhood. McDonald’s Omega indicated good internal consistency (ω = .878).

**Demographic characteristics.** We measured sex, age, ethnic group, and employment/student status. We also assessed BMI calculated in kg/m^2^ by measuring participants’ height and weight during the screening session.

**Individual difference measures**

With the exception of perceived food insecurity, which was categorically coded (further detail below), all individual difference measures were continuous, with higher scores indicating higher levels of each individual difference measure unless otherwise stated. Where appropriate, McDonald’s Omega (ω) was calculated as a measure of internal consistency.

**Impulsivity*.*** The 30-item Barratt Impulsiveness Scale was used to measure self-reported trait impulsivity (9). Participants rated questions (e.g. “I say things without thinking”) from 1 (rarely/never) to 4 (almost always/always). After reverse scoring relevant items, items from the Barratt Impulsiveness Scale were summed. The higher the summed score for all items, the higher the level of impulsiveness (min. = 30, max. = 120). We also examined each subscale (attentional, motor, non-planning). McDonald’s Omega was calculated as an indicator of internal consistency overall and for each subscale, with good internal consistency overall (ω =.857), and for attentional (ω =.740), and non-planning (ω =.741) subscales, and average internal consistency for motor (ω =.581) subscale.

**Inhibition*.*** A Stroop task (10), was used to measure inhibition. Participants were given colour words and asked to indicate the colour the word was printed in (not its meaning) by key press as quickly and accurately as possible. Trials were either congruent (word and its colour are the same), incongruent (word and its colour not the same), or control (coloured rectangles with no word). The task included 4 colours (red, green, blue, black) x 3 colour-stimuli congruency (congruent, incongruent, control) x 7 repetitions = 84 trials randomly sampled. For correct responses, the median reaction times (RTs) in incongruent and congruent trials was calculated (11, 12). The difference in median RTs between incongruent vs congruent trials was calculated [incongruent RT – congruent RT], giving the Stroop interference effect. Higher scores indicate poorer response inhibition (greater interference).

**Food choice motives.** The ‘Health’ (6 items, e.g. “Keeps me healthy”) and ‘Weight control’ (3 items, e.g. “Is low in calories”) subscales from the Food Choice Questionnaire (13) were used to assess food choice motives. Participants were asked to rate how important each statement is for their food choices and behaviours from 1 (Not at all important) to 4 (Very important). Both health and weight control scores were computed by averaging ratings for individual items of each dimension (health motivation: 6 items; weight control motivation: 3 items). Higher scores indicate stronger motives around health and weight control respectively. McDonald’s Omega indicated good internal consistency for health (ω = .774) and weight control (ω = .672) motives, and overall (ω =.751).

**Satiety responsiveness.** The 4-item satiety responsiveness subscale of the Adult Eating Behaviour Questionnaire (AEBQ) was used to measure satiety responsiveness (14). Participants rated questions (e.g., “I often get full before my meal is finished”) from 1 (strongly disagree) to 5 (strongly agree). Responses were averaged, with higher scores indicating a higher satiety responsiveness. McDonald’s Omega indicated good internal consistency (ω = .723).

**Plate clearing tendencies.** The 5-item plate clearing tendencies measure developed by Robinson and colleagues (15) was used to assess plate clearing tendencies. Participants rated questions (e.g. “I always tend to clear my plate when eating”) from 1 (Strongly disagree) to 5 (Strongly agree). Responses were averaged, with higher scores indicating higher plate clearing tendencies. McDonald’s Omega indicated good internal consistency (ω = .816).

**Perceived food insecurity*.*** The 6-item short-form version of the USDA 12-month Food Security Scale Questionnaire, one of the most widely used self-report questionnaires to measure food insecurity (16), was used to assess perceived food insecurity. Given the high volume of questionnaire measures in this study, we opted to use the short-form version as this has been found to have reasonable sensitivity and specificity as compared to the full 18-item (17) with less burden to the participant. Participants answered questions (e.g., “The food that (I/we) bought just didn't last, and (I/we) didn't have money to get more”) and based on the authors’ recommendations (18), responses to the 6 questions about food insecurity were coded as affirmatives (i.e. “yes”, “often true”, “sometimes true”, “almost every month”, “some months but not every month”) and negatives (i.e. “no”, “never true”, “only 1 or 2 months”). Coding of food security was based on the number of affirmatives as follows: 0-1 = “food secure”, 2-4 = “food insecure without hunger”, 5-6 = “food insecure with hunger”. The responses “don’t know” and blank responses reflected missing data, which we planned to impute following best practice guidance (18), but given very low incidences of “don’t know” responses and that imputing responses would not change categorisation, we did not impute data.

**Perceived ‘normal’ portion sizes*.*** A short computer-based task programmed in Inquisit was used to estimate perceived ‘normal’ portion sizes. Participants were asked to select the portion sizes they thought were a normal amount to eat for each of the portion-manipulated meals. They were asked to scroll through all images before making their selection, and indicate their choice using key press. Participants viewed images of all portion-manipulated meals in 40kcal increments from 0-1000kcal. For each participant we calculated the average selected portion size (kcal) across all portion-manipulated dishes.

**Compensatory health beliefs*.*** A 7-item questionnaire was used to assess compensatory health beliefs (adapted from (19, 20)). This included the 3-item weight-regulation subscale developed by Knäuper and colleagues (19), with participants rating questions (e.g. “Eating dessert can be made up for by skipping the main dish”) from 1 (not at all) to 5 (very much) on how much the compensatory health belief matched their own belief around weight control. This also included the 4-item portion size subscale developed by Poelman and colleagues (20), with participants rating questions (e.g. “If I eat a small meal, it’s fine to have a larger portion during the next meal”) from 1 (not at all) to 5 (very much) on how much the compensatory health belief matched their own belief around portion size. Responses were averaged for weight-regulation and portion size separately, with higher scores indicating stronger compensatory health beliefs. McDonald’s Omega indicated good internal consistency (ω = .806), so scores across the two measures (weight regulation and portion size) were averaged for analysis.

**Outcome measures**

**Daily energy intake.** All the foods served in the lab were weighed before and after breakfast/lunch/dinner/dessert in order to determine how much of each food had been consumed by the participants. Participants took photos of their snack box before bed and sent it to the research team via email. They were told that all items must be photographed, including uneaten items as well as empty wrappers (e.g., if they have eaten a chocolate bar, the empty wrapper must be photographed). Participants were asked to return the snack box including all food and wrappers the following day during the drop-in session. Returned items were checked against the photo in case any items were missing on return, but uneaten the night before (indicating they may have been eaten the following day or kept by the participant to eat later – in these cases this was checked with the participant).

Energy intake from study foods was calculated by multiplying the consumed weight of each food by the energy density (kcal/g) provided by the food manufacturer. Energy intake from additional self-reported food was estimated using intake24, a dietary recall system validated against interviewer-led recall (21, 22) (researcher entered in Study 1; participant entered in the laboratory during Study 2).

**Filler measures.** Participants completed mood ratings before and after eating each meal (on visual analogue scales ranging from 0 – 100 with embedded ‘hunger and fullness’ ratings) and a daily sleep quality questionnaire to distract from the focus on food intake and bolster the cover story that we were investigating the inteplay between mood, diet, and sleep.

Participants also completed a 1-minute computerised lexical decision task assessing categorisation of words during the lunch session each day. The task was introduced as “*A new way to assess mood by measuring speed of responding to positive versus neutral words*”. Five ‘positive’ mood-related words (e.g. “joyful”), 5 ‘neutral’ words (e.g. “pencil”), and 5 ‘non-words’ (e.g. “spraw”) were presented on the computer screen one by one in a random order. Participants were asked to respond to each word/non-word by pressing the left or right key marked on the keyboard to indicate whether the task displays a word or non-word (according to the key assignment specified in the task instructions). There were two different versions of the task, each consisting of different word sets – one for each study day.

**Additional results**

**Sample characteristics**

In Study 1 an independent samples t test revealed no evidence of a difference in age between lower SEP (M = 46.36, SD = 18.35) and higher SEP (M = 38.20, SD = 19.88) groups (p = .138). Visual inspection of the distribution of age indicated skew towards younger participants, and a non-parametric Mann-Whitney-U test also revealed no evidence of a difference in age between lower SEP (Mdn = 49, IQR = 21) and higher SEP (Mdn = 28, IQR = 34) groups (p = .159). An independent-samples t test (unequal variance assumed) revealed no evidence of a difference in BMI between lower SEP (26.50kg/m^2^, SD = 3.66) and higher SEP (M = 25.17kg/m^2^, SD = 2.07), p = .125. In Study 2, there were no baseline demographic differences, with an independent samples t test revealing no evidence of a difference in age between lower SEP (M = 50.00, SD = 17.74) and higher SEP (M = 53.13, SD = 13.28) groups, p = .502, and a further independent samples t test revealing no evidence of a difference in BMI between lower SEP (M = 27.65kg/m^2^, SD = 4.83) and higher SEP (M = 28.30kg/m^2^, SD = 5.69) groups, p = .680.

For full education equivalents see Table S4.

**Table S4. Educational equivalents**

| **Highest educational qualification (achieved or working towards)** | **Equivalent** |
| --- | --- |
| No formal qualifications | - |
| 1-3 GCSEs or equivalent – *US equivalent: High School Diploma/GED Certificate* | *Level 1: first certificate; GCSE – grades 3, 2, 1 or grades D, E, F, G; level 1 award; level 1 certificate; level 1 diploma; level 1 ESOL; level 1 essential skills; level 1 functional skills; level 1 national vocational qualification (NVQ); music grades 1, 2 and 3* |
| 4+ GCSEs or equivalent *– US equivalent: High School Diploma/GED Certificate* | *Level 2: CSE – grade 1; GCSE – grades 9, 8, 7, 6, 5, 4 or grades A*, A, B, C; intermediate apprenticeship; level 2 award; level 2 certificate; level 2 diploma; level 2 ESOL; level 2 essential skills; level 2 functional skills; level 2 national certificate; level 2 national diploma, level 2 NVQ; music grades 4 and 5; O level – grade A, B or C* |
| A level or equivalent – *US equivalent: Advanced Placement* | *Level 3: A level; access to higher education diploma; advanced apprenticeship; applied general; AS level; international Baccalaureate diploma; level 3 award; level 3 certificate; level 3 diploma; level 3 ESOL; level 3 national certificate; level 3 national diploma; level 3 NVQ; music grades 6, 7 and 8; tech level* |
| Certificate of higher education (CertHE) or equivalent – *US equivalent: Associate degree* | *Level 4: certificate of higher education (CertHE); higher apprenticeship; higher national certificate (HNC); level 4 award; level 4 certificate; level 4 diploma; level 4 NVQ* |
| Diploma of higher education (DipHE) or equivalent | *Level 5: diploma of higher education (DipHE); foundation degree; higher national diploma (HND); level 5 award; level 5 certificate; level 5 diploma; level 5 NVQ* |
| Bachelor or equivalent | *Level 6: degree apprenticeship; degree with honours – for example bachelor of the arts (BA) hons, bachelor of science (BSc) hons; graduate certificate; graduate diploma; level 6 award; level 6 certificate; level 6 diploma; level 6 NVQ; ordinary degree without honours* |
| Master’s degree or equivalent | *Level 7: integrated master’s degree, for example master of engineering (Meng); level 7 award; level 7 certificate; level 7 diploma; level 7 NVQ; master’s degree, for example master of arts (MA), master of science (MSc); postgraduate certificate; postgraduate certificate in education (PGCE); postgraduate diploma* |
| Doctorate or equivalent | *Level 8: doctorate, for example doctor of philosophy (PhD or Dphil); level 8 award; level 8 certificate; level 8 diploma* |

**Sensitivity analyses**

**Student status.** In Study 1, given the imbalance of student status across SEP groups (i.e. number of current university students in the higher SEP group) and the hypothesis that this might have been driving SEP differences in energy intake, two mixed ANOVA adjusting for student status were conducted. First, we ran a mixed ANOVA predicting total daily energy intake (primary outcome measure), which revealed a main effect of portion size (F(1,46) = 18.612, p<.001, partial eta 2 = .288), with 278.02 kcal less eaten on smaller portion size days (95% CI: 148.31, 407.74), a main effect of student status (F(1,46) = 8.06, p = .007, partial eta 2 = .149), with students eating 525.7kcal more than non-students (95% CI: 153.00, 898.4), no main effect of SEP (F(1,46) = 2.09, p = .155, partial eta 2 = .043), no interaction between portion size and SEP (F(1,46) = 1.351, p = .251, partial eta 2 = .029), and no interactions involving student status (ps > .087).

Second, we ran a mixed ANOVA predicting energy intake from non-manipulated foods (secondary outcome measure), which revealed no main effect of portion size (F(1,46) = 0.001, p = .970 partial eta^2^ < .001), a main effect of student status (F(1,46) = 7.268, p = .010, partial eta 2 = .136), with students eating 436.24kcal more than non-students (95% CI: 110.51, 761.96), no main effect of SEP (F(1,46) = 3.607, p = .064, partial eta^2^ = .073), no interaction between portion size and SEP (F(1,46) = 1.507, p = .226, partial eta^2^ = .032), and no other interactions with student status (ps > .192). Therefore, these analyses suggest that the main effects of SEP on energy intake outcomes observed in Study 1 (higher energy intake in higher SEP for daily and non-manipulated foods) was largely driven by the higher SEP participants currently studying in university education. Moreover, the lack of interaction between portion size condition and SEP on energy intake outcomes remained after accounting for current student status.

**Removing study aim guessers.** Participants were generally unaware of the aim of the studies. In Study 1, 1 participant guessed the aim of the study and in Study 2, 2 participants guessed the aim of the study. In Study 1, a total of 15 participants guessed the aim directly, guessed that portion size was related to the aims, or guessed we were measuring energy intake. In Study 2, a total of 7 guessed the aim directly, guessed that portion size was related to the aims, or guessed that we were measuring energy intake. As specified in our pre-registration, we retained aim-guessers for all primary analyses. Unless otherwise stated, the inclusion of these participants did not alter the pattern of findings for the primary analyses. In Study 1, we repeated the primary analysis after removing participants who guessed the study aim (n = 1), which was consistent with the primary analysis, revealing a main effect of portion size (F(1,47) = 20.057, p < .001, partial eta^2^ = .299), a main effect of SEP (F(1,47) = 7.034, p = .011, partial eta^2^ = .130), and no significant interaction between portion size and SEP (F(1,47) = 0.428, p = .516, partial eta^2^ = .009). In Study 2, we repeated the primary analysis after removing participants who guessed the study aim (n = 2), which was consistent with the primary analysis, revealing a main effect of portion size (F(1,42) = 5.249, p = .027, partial eta^2^ = .111), no main effect of SEP (F(1,42) = 0.099, p = .754, partial eta^2^ = .002), and no significant interaction between portion size and SEP (F(1,42) = 0.686, p = .412, partial eta^2^ = .016).

**Removing outliers.** No outliers on the primary outcome measure were identified in Study 1 or 2.

**Order effects.** We ran sensitivity analyses by repeating the primary analysis after adjusting for order that conditions were received by participants [smaller first vs larger first]. Findings were consistent with primary analyses. In Study 1**,** there was a main effect of portion size (F(1,46) = 20.695, p < .001, partial eta^2^ = .310), a main effect of SEP (F(1,46) = 7.307, p = .010, partial eta^2^ = .137), no interaction between portion size and SEP (F(1,46) = 0.514, p = .477, partial eta^2^ = .011), and no main effect of order (F(1,46) = 0.045, p = .833, partial eta^2^ = .001), nor any interactions with meal order (ps>.110). In Study 2, there was a main effect of portion size (F(1,42) = 5.595, p = .023), no main effect of SEP (F(1,42) = 0.02, p = .888), no interaction between portion size and SEP (F(1,42) = 0.755, p = .390), and no main effect of order (F(1,42) = 1.132, p = .293), nor any interactions with meal order (ps>.780).

**SEP.** We ran a sensitivity analysis by repeating the primary analyses after replacing the primary SEP indicator (Study 1: highest educational qualification [lower vs higher]; Study 2: SSS [lower vs higher]) with alternative SEP indicators. Results were consistent with the primary analyses, with no evidence of an interaction between portion size and SEP on energy intake. See Table S6 for full ANOVA results.

**Secondary analyses**

**Individual difference measures.** We planned to explore whether any of the individual difference measures mediated potential socioeconomic differences in susceptibility to the PSE. However, in the absence of moderation by SEP in Study 1 or 2, we repeated the primary analysis replacing SEP with each individual difference measure, to examine whether any of the individual difference measures moderated the main effect of portion size on daily energy intake. As planned, we instead ran several mixed ANOVA including portion size, each individual difference measure, and their interaction, predicting total daily energy intake. There was no evidence that variation in any individual difference measure interacted with the portion size effect on total daily energy intake, for full ANOVA results see Table S5.

**Physical activity.** To assess compliance with the physical activity restriction, a mixed ANOVA was used to test the effect of portion size (smaller, larger), SEP (lower, higher), and portion size*SEP on number of minutes of moderate-to-vigorous physical activity (MVPA). In Study 1, there was no main effect of portion size on MVPA (F(1,47) = 0.954, p = .334, partial Eta2 = .020), no main effect of SEP (F(1,47) = 0.006, p = .941, partial Eta2 < .001), and no interaction (F(1,47) = 0.006, p = .941, partial Eta2 < .001). In Study 2, there was no main effect of portion size on MVPA (F(1,42) = 0.936, p = .339, partial Eta2 = .022), no main effect of SEP (F(1,42) = 4.059, p = .050, partial Eta2 = .088), and no interaction (F(1,42) = 0.035, p = .852, partial Eta2 = .001).

**Normality, Familiarity, Liking.** Independent samples t tests were conducted to compare higher and lower SEP groups on portion size normality, familiarity and liking of portion-manipulated foods; see Table S7 for summary normality, familiarity and liking ratings split by SEP group. In Study 1, there was no evidence that lower vs higher SEP groups differed in terms of the perceived normality of smaller lunch portions (t(37.94) = 1.36, p = .183), larger lunch portions (t(48) = 1.04, p = .305), smaller dinner portions (t(48) = 1.14, p = .262), or larger dinner portions (t(48) = 0.93, p = .355). In Study 2, there was no evidence that lower vs higher SEP groups differed in terms of the perceived normality of smaller breakfast portions (t(44) = 1.55, p = .128), larger breakfast portions (t(44) = 0.753, p = .455), smaller lunch portions (t(44) = -1.02, p = .316)), larger lunch portions (t(44) = -0.716, p = .478)), smaller dinner portions (t(44) = -1.95, p = .057)), or larger dinner portions (t(44) = -0.517, p = .608).

In Study 1 there was no evidence that lower vs higher SEP groups differed in terms of liking of pasta bake (t(48) = 1.32, p = .192) or beef chilli with rice (t(48) = 1.51, p = .139), or in terms of familiarity of pasta bake (t(48) = -0.47, p = .639) or beef chilli with rice (t(48) = 0.28, p = .783). In Study 2, there was no evidence that lower vs higher SEP groups differed in terms of liking of toast (t(42) = 0.734, p = .467), cornflakes (t(37.37) = 0.921, p = .363), yoghurt (t(32) = 0.475, p = .638), pasta bake (t(43) = -0.907, p = .369), or vegetarian chilli with rice (t(44) = -1.794, p = .08). There was no evidence that lower vs higher SEP groups differed in terms of familiarity of toast (t(44) = 0.138, p = .891), cornflakes (t(42) = 1.54, p = .13), yoghurt (t(40) = -0.231, p = .819), pasta bake (t(43) = -0.346, p = .731), or vegetarian chilli with rice (t(44) = -0.899, p = .373).

**Portion size manipulation awareness.** In Study 1, 62% (n = 31) reported that they noticed the difference in portion sizes between the conditions. Among those who reported noticing a difference, 70.9% (n = 22) correctly identified the portions received on each day, which was above chance expectation (χ^2^(1) = 5.45, p = .020). There was no evidence that the tendency to report noticing a difference and accurately identifying the portions received on each day differed by SEP (χ^2^(1) < .001, p > .99). In Study 2, 74% (n = 34) reported that they noticed the difference in portion sizes between the conditions. Among those who reported noticing a difference, 50% (n = 17) correctly identified the portions received on each day, which was not above chance expectation(χ^2^(1) < .001, p > .99). There was no evidence that the tendency to report noticing a difference and accurately identifying the portions received on each day differed by SEP (χ^2^(1) = 0.093, p = .760). See Table S8 and S9.

**Table S5.** Secondary analysis - mixed ANOVA results predicting total daily energy intake (kcal): portion size, individual difference measure, and portion size * individual difference measure

|  | **Study 1** | | | **Study 2** | | |
| --- | --- | --- | --- | --- | --- | --- |
| ***Individual difference measure*** | **Main effect portion size** | **Main effect ID measure** | **Interaction** | **Main effect portion size** | **Main effect ID measure** | **Interaction** |
| **Impulsivity** | F(1,48) = 0.461, p = .50, partial Eta^2^ = .01 | F(1,48) = 1.29, p = .262, partial Eta^2^ = .026 | F(1,48) = 0.903, p = .903, partial Eta^2^ < .001 | F(1,44) = 0.695, p = .409, partial Eta^2^ = .016 | F(1,44) = 1.324, p = .256, partial Eta^2^ = .029 | F(1,44) = 1.631, p = .208, partial Eta^2^ = .036 |
| **Inhibition** | F(1,48) = 10.47, p = .002, partial Eta^2^ = .182 | F(1,48) = 0.254, p = .617, partial Eta^2^ = .005 | F(1,48) = 0.346, p = .559, partial Eta^2^ = .007 | F(1,44) = 0.775, p = .383, partial Eta^2^ = .017 | F(1,44) = 3.322, p = .075, partial Eta^2^ = .070 | F(1,44) = 1.040, p = .313, partial Eta^2^ = .023 |
| **Health food choice motives** | F(1,48) = 0.116, p = .735, partial Eta^2^ = .002 | F(1,48) = 2.0, p = .164, partial Eta^2^ = .040 | F(1,48) = 1.366, p = .248, partial Eta^2^ = .028 | F(1,44) < .001, p = .988, partial Eta^2^ < .001 | F(1,44) = 0.759, p = .388, partial Eta^2^ = .017 | F(1,44) = 0.192, p = .664, partial Eta^2^ = .004 |
| **Weight control food choice motives** | F(1,48) = 0.263, p = .611, partial Eta^2^ = .005 | F(1,48) = 0.347, p = .559, partial Eta^2^ = .007 | F(1,48) = 0.440, p = .510, partial Eta^2^ = .009 | F(1,44) = 0.081, p = .777, partial Eta^2^ = .002 | F(1,44) = 0.125, p = .725, partial Eta^2^ = .003 | F(1,44) = 1.119, p = .296, partial Eta^2^ = .025 |
| **Satiety responsiveness** | F(1,48) = 1.848, p = .180, partial Eta^2^ = .037 | F(1,48) = 17.67, p < .001, partial Eta^2^ = .269 | F(1,48) = 0.002, p = .967, partial Eta^2^ < .001 | F(1,44) = 0.380, p = .541, partial Eta^2^ = .009 | F(1,44) = 13.28, p < .001, partial Eta^2^ = .232 | F(1,44) = 1.852, p = .180, partial Eta^2^ = .040 |
| **Plate-clearing tendencies** | F(1,48) = 1.001, p = .322, partial Eta^2^ = .020 | F(1,48) = 15.11, p < .001, partial Eta^2^ = .239 | F(1,48) = 0.006, p = .939, partial Eta^2^ < .001 | F(1,44) = 3.939, p = .053, partial Eta^2^ = .082 | F(1,44) = 5.90, p = .019, partial Eta^2^ = .118 | F(1,44) = 1.974, p = .167, partial Eta^2^ = .043 |
| **Perceived food insecurity** | F(1,46) = 4.54, p = .038, partial Eta^2^ = .088 | F(1,46) = 0.201, p = .818, partial Eta^2^ = .008 | F(1,46) = 1.386, p = .260, partial Eta^2^ = .056 | F(1,42) = 4.416, p = .042, partial Eta^2^ = .095 | F(1,42) = 0.495, p = .688, partial Eta^2^ = .034 | F(1,42) = 0.860, p = .469, partial Eta^2^ = .058 |
| **Compensatory health beliefs** | F(1,48) = 1.395, p = .243, partial Eta^2^ = .028 | F(1,48) = 1.457, p = .233, partial Eta^2^ = .029 | F(1,48) = 0.126, p = .724, partial Eta^2^ = .003 | F(1,44) = 4.082, p = .049, partial Eta^2^ = .085 | F(1,44) = 0.273, p = .604, partial Eta^2^ = .006 | F(1,44) = 1.670, p = .203, partial Eta^2^ = .037 |
| **Perceived normal portion (kcal)** | F(1,48) = 3.904, p = .054, partial Eta^2^ = .075 | F(1,48) = 7.29, p = .010, partial Eta^2^ = .132 | F(1,48) = 0.289, p = .593, partial Eta^2^ = .006 | F(1,44) = 1.071, p = .306, partial Eta^2^ = .024 | F(1,44) = 5.068, p = .029, partial Eta^2^ = .103 | F(1,44) = 3.441, p = .070, partial Eta^2^ = .073 |
| **BMI (kg/m^2^)** | F(1,48) = 0.196, p = .660, partial eta^2^ = .004 | F(1,48) = 0.276, p = .602, partial eta^2^ = .006 | F(1,48) = 0.976, p = .328, partial eta^2^ = .020 | F(1,44) = 1.240, p = .272, partial eta^2^ = .027 | F(1,44) = 0.276, p = .602, partial eta^2^ = .006 | F(1,44) = 0.471, p = .496, partial eta^2^ = .011 |

**Table S6.** Sensitivity analysis - mixed ANOVA results predicting total daily energy intake (kcal): portion size, alternative SEP indicator, and portion size * SEP indicator

|  | **Study 1** | | | **Study 2** | | |
| --- | --- | --- | --- | --- | --- | --- |
| ***Alternative SEP indicator*** | **Main effect portion size** | **Main effect SEP** | **Interaction** | **Main effect portion size** | **Main effect SEP** | **Interaction** |
| **Level of education (continuous score)** | F(1,48) = 22.00, p < .001, partial Eta^2^ = .314 | F(1,48) = 4.373, p = .042, partial Eta^2^ = .083 | F(1,48) = 0.791, p = .378, partial Eta^2^ = .016) | F(1,44) = 5.756, p = .021, partial Eta^2^ = .116 | F(1,44) = 1.614, p = .211, partial Eta^2^ = .035 | F(1,44) = 0.004, p = .952, partial Eta^2^ <.001 |
| **SSS (continuous score)** | F(1,48) = 0.26, p = .613, partial Eta^2^ = .005 | F(1,48) = 0.288, p = .594, partial Eta^2^ = .006 | F(1,48) = 0.426, p = .517, partial Eta^2^ = .009 | F(1,44) = 4.135, p = .048, partial Eta^2^ = .086 | F(1,44) = 0.198, p = .659, partial Eta^2^ = .004 | F(1,44) = 1.693, p = .200, partial Eta^2^ = .037 |
| **Equivalised income** | F(1,48) = 2.382, p = .130, partial Eta^2^ = .052 | F(1,48) = 1.068, p = .307, partial Eta^2^ = .024 | F(1,48) = 1.326, p = .256, partial Eta^2^ = .030 | F(1,37) = 2.071, p = .159, partial Eta^2^ = .053 | F(1,37) = 2.533, p = .120, partial Eta^2^ = .064 | F(1,37) = 0.009, p = .924, partial Eta^2^ <.001 |
| **Self-reported childhood financial hardship** | F(1,48) = 10.071, p = .003, partial Eta^2^ = .173 | F(1,48) = 10.064, p = .003, partial Eta^2^ = .173 | F(1,48) = 1.157, p = .287, partial Eta^2^ = .024 | F(1,44) = 1.080, p = .304, partial Eta^2^ = .024 | F(1,44) = 1.290, p = .262, partial Eta^2^ = .028 | F(1,44) = 0.002, p = .968, partial Eta^2^ < .001 |
| **Highest educational qualification (lower vs higher)^a^** | - | - | - | F(1,44) = 5.895, p = .019, partial Eta^2^ = .118 | F(1,44) = 0.886, p = .352, partial Eta^2^ = .020 | F(1,44) = 0.255, p = .616, partial Eta^2^ =.006 |

Notes. SEP = Socioeconomic position. ^a^ Study 2 only.

**Table S7. Mean (SD) Normality, Familiarity and Liking ratings of all portion-manipulated study foods**

|  |  | Study 1 | | | Study 2 | | |
| --- | --- | --- | --- | --- | --- | --- | --- |
|  |  | Higher SEP | Lower SEP | Overall | Higher SEP | Lower SEP | Overall |
| Normality | **Smaller portion** | | | | | | |
|  | Toast, Cereal & Yoghurt | - | - | - | 5.00 (1.00) | 5.43 (0.90) | 5.22 (0.96) |
|  | Pasta bake | 3.52 (0.77) | 3.76 (0.44) | 3.64 (0.63) | 4.22 (0.60) | 4.04 (0.56) | 4.13 (0.58) |
|  | Chilli with rice^a^ | 4.24 (0.83) | 4.48 (0.65) | 4.36 (0.75) | 4.52 (0.73) | 4.13 (0.63) | 4.33 (0.70) |
|  | **Larger portion** | | | | | | |
|  | Toast, Cereal & Yoghurt | - | - | - | 6.35 (0.83) | 6.52 (0.73) | 6.43 (0.78) |
|  | Pasta bake | 5.32 (1.15) | 5.64 (1.04) | 5.48 (1.09) | 5.30 (1.02) | 5.09 (1.04) | 5.20 (1.03) |
|  | Chilli with rice^a^ | 5.32 (1.07) | 5.56 (0.71) | 5.44 (0.91) | 5.83 (0.89) | 5.70 (0.82) | 5.76 (0.85) |
| Liking | Toast | - | - | - | 5.52 (1.68) | 5.86 (1.31) | 5.68 (1.51) |
|  | Cereal | - | - | - | 4.41 (1.92) | 4.89 (1.37) | 4.63 (1.69) |
|  | Yoghurt | - | - | - | 5.11 (1.91) | 5.44 (2.10) | 5.26 (1.97) |
|  | Pasta bake | 5.16 (1.55) | 5.68 (1.22) | 5.42 (1.40) | 5.35 (1.56) | 4.91 (1.69) | 5.13 (1.62) |
|  | Chilli with rice^a^ | 5.40 (1.50) | 5.96 (1.09) | 5.68 (1.33) | 5.83 (1.37) | 5.04 (1.58) | 5.43 (1.52) |
| Familiar | Toast | - | - | - | 4.96 (2.14) | 5.04 (1.14) | 5.00 (2.12) |
|  | Cereal | - | - | - | 3.17 (2.41) | 4.24 (2.14) | 3.68 (2.32) |
|  | Yoghurt | - | - | - | 5.55 (2.04) | 5.40 (2.04) | 5.48 (2.02) |
|  | Pasta bake | 4.76 (1.72) | 4.52 (1.87) | 4.64 (1.78) | 4.61 (1.78) | 4.41 (2.09) | 4.51 (1.91) |
|  | Chilli with rice^a^ | 4.92 (1.96) | 5.08 (2.12) | 5.00 (2.02) | 4.74 (1.84) | 4.22 (2.09) | 4.48 (1.96) |

**Note.** SEP = Socioeconomic position. Liking rated from 1 (Not at all) to 7 (Very much); Familiarity from 1 (Strongly disagree) to 7 (Strongly agree) with the statement “I would normally eat this type of food”; Normality rated from 1 (Not normal, far too small) to 7 (Not normal, far too large), with a midpoint of 4 (Normal). Dashes represent dishes that were not included as portion-manipulated meals. ^a^ Note beef chilli in Study 1 and vegetarian chilli in Study 2. In Study 2 an additional response option was provided (“N/A did not eat”).

**Table S8**. Counts of participants who reported noticing a difference in portion size (yes/no) and who guessed the portion sizes of each meal correctly (yes/no)

|  |  | Study 1 | | | Study 2 | | |
| --- | --- | --- | --- | --- | --- | --- | --- |
|  |  | Reported noticing difference in portion sizes | | | Reported noticing difference in portion sizes | | |
|  |  | No | Yes | Total | No | Yes | Total |
| Guessed portions correct | No | 13 | 9 | 22 | 11 | 16 | 27 |
|  | Yes | 6 | 22 | 28 | 2 | 17 | 19 |
|  | Total | 19 | 31 | 50 | 13 | 33 | 46 |

|  |  | Study 1 | | Study 2 | |
| --- | --- | --- | --- | --- | --- |
|  |  | SEP | | SEP | |
|  |  | High | Low | High | Low |
| Reported noticing and guessed portions correct | Yes | 11 | 11 | 8 | 9 |
|  | No | 14 | 14 | 15 | 14 |

**Table S9**. Counts of participants who reported noticing a difference in portion size and accurately guessed the portion sizes split by SEP group

Note. SEP = Socioeconomic position.

**References**

1. NHS Digital. “Health Survey for England, 2014”. Department of Epidemiology and Public Health University College London, 2016.

2. HESA. Who's studying in HE? 2022. Available from: https://www.hesa.ac.uk/data-and-analysis/students/whos-in-he

3. Haynes A, Hardman CA, Halford JCG, Jebb SA, Mead BR, Robinson E. Reductions to main meal portion sizes reduce daily energy intake regardless of perceived normality of portion size: a 5 day cross-over laboratory experiment. International Journal of Behavioral Nutrition & Physical Activity. 2020;17(1):1-13. doi: 10.1186/s12966-020-0920-4. PubMed PMID: 141726154.

4. Giatti L, Camelo LdV, Rodrigues JFdC, Barreto SM. Reliability of the MacArthur scale of subjective social status - Brazilian Longitudinal Study of Adult Health (ELSA-Brasil). BMC public health. 2012;12(1):1096-. doi: 10.1186/1471-2458-12-1096.

5. Office for National Statistics. Chapter 3: Equivalised income. UK: Compend Fam Spend. 2015.

6. Griskevicius V, Delton AW, Robertson TE, Tybur JM. Environmental Contingency in Life History Strategies: The Influence of Mortality and Socioeconomic Status on Reproductive Timing. Journal of personality and social psychology. 2011;100(2):241-54. PubMed PMID: edscal.23864190.

7. Maner JK, Dittmann A, Meltzer AL, McNulty JK. Implications of life-history strategies for obesity. Proceedings of the National Academy of Sciences of the United States of America. 2017;114(32):8517-22. PubMed PMID: edsjsr.26486915.

8. Hill SE, Prokosch ML, DelPriore DJ, Griskevicius V, Kramer A. Low Childhood Socioeconomic Status Promotes Eating in the Absence of Energy Need. Psychological Science. 2016;27(3):354-64. PubMed PMID: edsjsr.24763482.

9. Patton JH, Stanford MS, Barratt ES. Factor structure of the Barratt Impulsiveness Scale. Journal of clinical psychology. 1995;51(6):768-74. PubMed PMID: edscal.2945939.

10. Jensen AR, Rohwer Jr WD. The stroop color-word test: A review. Acta Psychologica. 1966;25(C):36-93. doi: 10.1016/0001-6918(66)90004-7. PubMed PMID: edselc.2-52.0-0013874520.

11. Ratcliff R, editor Methods for Dealing With Reaction Time Outliers1993 1993/01/01/; United States: APA American Psychological Association.

12. Rand RW, Guillaume AR. Reaction Times and other Skewed Distributions. Linnaeus University; 2020.

13. Steptoe A, Pollard TM, Wardle J. Development of a measure of the motives underlying the selection of food - the food choice questionnaire. Appetite. 1995;25(3):267-84.

14. Hunot C, Fildes A, Croker H, Llewellyn CH, Wardle J, Beeken RJ. Appetitive traits and relationships with BMI in adults: Development of the Adult Eating Behaviour Questionnaire. 2016:356-63.

15. Robinson E, Aveyard P, Jebb SA. Is plate clearing a risk factor for obesity? A cross-sectional study of self-reported data in US adults. 2015:301-4.

16. Nettle D, Andrews C, Bateson M. Food insecurity as a driver of obesity in humans: The insurance hypothesis. Cambridge University Press (CUP); 2017.

17. Blumberg SJ, Bialostosky K, Hamilton WL, Briefel RR. The effectiveness of a short form of the household food security scale. American journal of public health (1971). 1999;89(8):1231-4. PubMed PMID: edscal.1914141.

18. Bickel G, Nord, M., Price, C., Hamilton, W., & Cook, J. Guide to measuring household food security. 2000.

19. Knäuper B, Rabiau M, Cohen O, Patriciu N. Compensatory health beliefs: scale development and psychometric properties. Psychology & Health. 2004;19(5):607-24. doi: 10.1080/0887044042000196737. PubMed PMID: 106578042. Language: English. Entry Date: 20050211. Revision Date: 20200708. Publication Type: Journal Article.

20. Poelman MP, Vermeer WM, Vyth EL, Steenhuis IHM. 'I don't have to go to the gym because I ate very healthy today': the development of a scale to assess diet-related compensatory health beliefs. Public health nutrition (Wallingford). 2013;16(2):267-73. PubMed PMID: edscal.27078754.

21. Simpson E, Bradley J, Poliakov I, Jackson D, Olivier P, Adamson AJ, et al. Iterative Development of an Online Dietary Recall Tool: INTAKE24. Nutrients. 2017;9(2):118. doi: 10.3390/nu9020118.

22. Bradley J, Simpson E, Poliakov I, Matthews J, Olivier P, Adamson A, et al. Comparison of INTAKE24 (an Online 24-h Dietary Recall Tool) with Interviewer-Led 24-h Recall in 11–24 Year-Old. MDPI AG; 2016.
